# Risperidone Reverses the Downregulation of BDNF in Hippocampal Neurons and MK801-Induced Cognitive Impairment in Rats

**DOI:** 10.3389/fnbeh.2019.00163

**Published:** 2019-07-23

**Authors:** Wenjuan Yu, Min Zhu, Hongwei Fang, Jie Zhou, Le Ye, Wenyu Bian, Yuan Wang, Hui Zhu, Jie Xiao, Hao Zhu, Huafang Li

**Affiliations:** ^1^Shanghai Mental Health Center, Shanghai Jiao Tong University School of Medicine, Shanghai, China; ^2^Department of Pharmacy, South Campus, Renji Hospital, Shanghai Jiao Tong University School of Medicine, Shanghai, China; ^3^Department of Anesthesiology and Intensive Care Unit, Dongfang Hospital, Tongji University, Shanghai, China; ^4^Department of Anesthesiology, Renji Hospital, Shanghai Jiao Tong University School of Medicine, Shanghai, China; ^5^Shanghai Key Laboratory of Psychotic Disorders, Shanghai, China; ^6^Clinical Research Center, Shanghai Jiao Tong University School of Medicine, Shanghai, China

**Keywords:** MK-801, schizophrenia, risperidone, BDNF, cognitive function

## Abstract

MK-801, also known as dizocilpine, is a non-competitive N-methyl-D-aspartic acid (NMDA) receptor antagonist that induces schizophrenia-like symptoms. Our previous study showed that brain-derived neurotrophic factor (BDNF) signaling was upregulated in cultured hippocampal astrocytes in response to MK-801. However, dysfunctional NMDA receptors are mainly expressed in neurons. The effects of MK-801 on neuron-derived BDNF expression and of risperidone on MK-801-induced cognitive impairment and changes in BDNF expression are unclear. To address this issue, we examined BDNF expression in the hippocampus of rats that received repeated injections of MK-801 (0.5 mg/kg body weight for 6 days) and in primary cultured hippocampal neurons incubated with 20 μM MK-801 for 24 h. BDNF expression and cognitive function were also evaluated in rats receiving intraperitoneal injections of risperidone (1 mg/kg body weight) once daily for 7 days and in hippocampal neurons incubated with 10 μM risperidone following MK801 treatment. MK-801 treatment decreased BDNF expression in the rat hippocampus as well as the expression and secretion of BDNF in hippocampal neurons *in vitro*. However, risperidone reversed the effects of MK801 on BDNF level and improved cognitive function in rats treated with MK801. These findings suggest that risperidone may alleviate cognitive impairment caused by MK801 *via* upregulation of BNDF signaling in the hippocampus.

## Introduction

Schizophrenia is a chronic and debilitating syndrome with an onset in adolescence and young adulthood followed by a chronic course, episodic aggravation, and often unfavorable outcome that affects approximately 1% of the population worldwide (Capuano et al., 2002; Jobe and Harrow, 2005). The N-methyl-D-aspartic acid receptor (NMDA-R) hypofunction hypothesis of schizophrenia suggests that NMDA-Rs play a central role in the etiology of the disorder (Olney and Farber, 1995). MK-801 (dizocilpine) is a non-competitive antagonist of NMDA-R with an extremely high affinity [10- to 100-fold higher than that of phencyclidine (PCP) or ketamine; Kornhuber and Weller, 1997] and selectivity for the PCP binding site on the receptor (Wong et al., 1986). Systemic administration of MK-801 induces schizophrenia-like symptoms in rodents (Deiana et al., 2015). Our previous study showed that repeated high doses (0.5 mg/kg every day for 6 days) of MK-801 in rats induced schizophrenia-like behavior (Yu et al., 2011).

Brain-derived neurotrophic factor (BDNF) is the most abundant neurotrophin in the brain and was originally identified as a neuronal survival factor (Barde et al., 1982) that promotes the proliferation and differentiation of neurons, affects the shape and number of dendritic spines—a critical determinant of neural information processing capacity (Cohen-Cory et al., 2010; Kuczewski et al., 2010)—and regulates synaptic plasticity in the hippocampus linked to learning and memory (Gottschalk et al., 1999). Age-related declines in BDNF signaling may contribute to memory deficits (Kuczewski et al., 2010; Zhang et al., 2012). By binding to TrkB and p75 neurotrophin (p75NTR) receptors, BDNF mediates long-term potentiation (LTP) and long-term depression (LTD), respectively, which are two forms of synaptic plasticity that represent cellular correlates of learning and memory in the hippocampus (Lu and Martinowich, 2008). Deficits in these processes may underlie some of the cognitive deficits exhibited by schizophrenia patients. Many studies have suggested that BDNF plays an important role in the pathophysiology of schizophrenia (Lu and Martinowich, 2008; Zhang et al., 2012). It is also thought that antipsychotics differentially affect serum or plasma BDNF protein levels according to the ethnic background of patients with schizophrenia (Huang, 2013).

Our previous study showed that BDNF signaling is upregulated in cultured hippocampal astrocytes in response to MK-801 (Yu W. et al., 2015). However, dysfunctional NMDA-Rs are mainly expressed in neurons. In the present study, we investigated the effect of MK-801 on neuron-derived BDNF expression in rats and in primary hippocampal neurons treated with MK801 as schizophrenia models. Risperidone is a second-generation antipsychotic drug (SGA) with high affinity for both serotonin 2A and dopamine D2 receptors and modest affinity for histamine and α2 adrenergic receptors (Kumari et al., 2015). Previous studies reported that cognitive impairment induced by repeated NMDA-R blockade can be reversed by SGAs but not first-generation antipsychotic drugs (Song et al., 2016; Liu et al., 2018). We also examined the effects of risperidone on MK-801-induced cognitive impairment and changes in BDNF expression.

## Materials and Methods

### Primary Hippocampal Neuron Culture and Drug Treatment

Hippocampal neurons were isolated as previously described (Li et al., 2015), with minor modifications. Briefly, pregnant Sprague–Dawley rats at embryonic day (E)17–E18 were anesthetized with sodium pentobarbital (100 mg/kg i.p.). The embryos were decapitated with sterile scissors and forceps under a dissecting microscope to expose the brain tissue. After careful removal of the meninges, the hippocampi were incubated in 0.05% trypsin (Sigma-Aldrich) for 10 min with gentle shaking and the reaction was terminated by adding 5 ml Dulbecco’s Modified Eagle’s Medium (DMEM) with 10% fetal bovine serum (FBS; Gibco). Dissociated cells were resuspended in DMEM (Gibco) supplemented with 10% FBS, 1 mM glutamine (Gibco), and 1% penicillin/streptomycin (Genom, Hangzhou, China) and seeded in 35-mm dishes pre-coated with 0.1 mg/ml poly-L-lysine (Sigma-Aldrich). After culturing at 37°C in a humidified atmosphere of 5% CO_2_ for 4 h, the medium was replaced with Neurobasal medium containing 2% B27 (Gibco), 1 mM glutamine, and 1% penicillin/streptomycin. After 2 days, 5 mg/ml cytosine arabinoside (Sigma-Aldrich) was added into the medium to prevent glial cell proliferation. Thereafter, half of the culture medium was replaced with fresh medium twice a week and neurons were used for experiments at 7–10 days *in vitro*.

Neurons were treated with 20 μM MK801 or left untreated for 24 h; cells and the culture supernatant were collected for detection of BDNF mRNA and protein. In the second set of experiments including four groups, neurons were treated with or without 20 μM MK801 for 2 h; the culture medium was replaced with fresh medium with or without 10 μM risperidone, followed by continuous incubation for 24 h, and cells and the supernatant were collected for analysis.

### Enzyme-Linked Immunosorbent Assay (ELISA)

BDNF concentration in the culture supernatant was measured using a commercially available enzyme-linked immunosorbent assay (ELISA) kit (BDNF Emax Immunoassay; Promega, Madison, WI, USA) according to the manufacturer’s instructions. Briefly, the monoclonal antibody was added to each well of a 96-well plate followed by overnight incubation at 4°C. The following reagents were then sequentially added to the wells: samples and BDNF standards in duplicate (with incubation for 2 h at room temperature); anti-human BDNF polyclonal antibody (with incubation for 2 h at room temperature); anti-IgY horseradish peroxidase (HRP; with incubation for 1 h at room temperature); and 3,3′,5,5′-tetramethylbenzidine solution (with incubation for 20 min at room temperature). The plate was washed with Tris-buffered saline (TBS) containing 0.05% Tween 20. A stop solution was added to each well and absorbance was measured at 450 nm with a microplate reader (Bio-Rad, Hercules, CA, USA) within 30 min. BDNF concentration was calculated based on a standard curve.

### Rat Model and Pharmacological Treatment

Male Sprague–Dawley rats (210–240 g, *n* = 40) from the Animal Care Facility at Shanghai Jiao Tong University School of Medicine were used for experiments. Rats were housed in pairs on a 12:12-h light/dark cycle and were allowed 1 week to acclimate to the housing conditions, with free access to food and water prior to the study.

Dizocilpine [MK-801; (5R,10S)-(+)-5-methyl-10,11-dihydro-5H-dibenzo(a,d) cyclohepten-5,10-imine] and risperidone were purchased from Sigma-Aldrich (St.Louis, MO, USA) and dissolved in normal saline. There were two animal cohorts in this study. The first comprised 16 rats (*n* = 8 each group) that were randomly assigned to the MK-801 or control group and received intraperitoneal injection (i.p.) of MK-801 (0.5 mg/kg body weight) or an equal volume of saline, respectively, once daily for 6 days. The second cohort comprised 24 rats that were randomly divided into control (saline), MK-801, and MK-801+ risperidone (MK801/Ris) groups (*n* = 8 each group). The rats were administered MK-801 (0.5 mg/kg body weight, i.p.) once daily for 6 days. Subsequently, rats in the MK-801+ risperidone group received risperidone (1 mg/kg body weight, i.p.) once daily for 7 days, whereas the MK-801-only group received an equal volume of normal saline. Control group received an equal volume of normal saline once daily for 13 days. All experimental procedures were in accordance with the guidelines of the Animal Care Committee of Laboratory Animals of Shanghai Jiao Tong University School of Medicine and the principles and guidelines of the USA National Institutes of Health Guide for the Care and Use of Laboratory Animals (Publication no. 80-23, revised in 1996). The animal protocols used in this work were evaluated and approved by the Animal Care Committee of Laboratory Animals at Shanghai Jiao Tong University School of Medicine.

### Immunohistochemistry

One hour after the last MK-801 injection, rats in the first cohort (*n* = 4 per group) were deeply anesthetized with sodium pentobarbital (100 mg/kg i.p.) and transcardially perfused with 100 ml saline solution followed by 400 ml of 4% paraformaldehyde in 0.01 M phosphate-buffered saline (PBS; pH 7.4). The brain was immediately removed, post-fixed for 2 h in the same fixative, and cryoprotected in 20% sucrose solution at 4°C for 24 h. Serial coronal sections were cut at a thickness of 15 μm at various levels (75-μm intervals) of the hippocampus on a freezing microtome (Leica, Wetzlar, Germany) and mounted on slides. Six sections were selected from the right or left hippocampus of each rat and were incubated in 3% hydrogen peroxide to quench endogenous peroxidase activity, then blocked and permeabilized in 0.01 M PBS with 1% bovine serum albumin (Gibco, Grand Island, NY, USA) and 0.3% Triton X-100 for 1 h at room temperature. The sections were incubated overnight at 4°C with anti-BDNF antibody (1:1,000, Sigma-Aldrich), followed by HRP-conjugated secondary antibody (Sigma-Aldrich) for 2 h at room temperature. Immunoreactivity was visualized by incubating the sections in a solution of 0.05% 3, 3-diaminobenzidine and 3% hydrogen peroxide in 0.01 M PBS. The specimens were dehydrated, mounted, and photographed using a Leica DM6000 microscope with a charge-coupled device 2/3 camera. We selected four random squares (300 × 300 μm) from each section for analysis. Images were converted to grayscale, and optical density (OD) was analyzed with RS Image Pro v.4.5 (Roper Scientific, Trenton, NJ, USA).

### Western Blotting

Rats (*n* = 4 per group) were deeply anesthetized with sodium pentobarbital (100 mg/kg i.p.) 1 h after the last MK-801 injection in the first cohort, and transcardially perfused with 100 ml saline solution. Hippocampi were immediately removed and lysed in buffer composed of 50 mM Tris–HCl, pH 7.5, 150 mM NaCl, 1% Nonidet P-40, 0.5% sodium deoxycholate, 0.1% sodium dodecyl sulfate (SDS), 1 mM EDTA, 1 mM sodium orthovanadate, 10 mM sodium fluoride, 4 μg/ml leupeptin, 1 μg/ml aprotinin, and 100 μg/ml phenylmethylsulfonyl fluoride (all from Sigma-Aldrich). After incubation on ice for 15 min, homogenates were clarified by centrifugation at 12,000× *g* for 10 min at 4°C and the supernatant was collected. Protein concentration was determined with the bicinchoninic acid assay. For western blotting, 20 μg protein per gel lane was electrophoresed on a 12% SDS/polyacrylamide gel and transferred to a nitrocellulose membrane that was blocked in a solution of 5% non-fat dry milk dissolved in TBS with 0.1% Tween 20 (TBS-T) and washed with TBS-T, then incubated overnight with antibodies against BDNF (1:2,000) and glyceraldehyde 3-phosphate dehydrogenase (GAPDH; 1:5,000; both from Sigma-Aldrich). The next day, the membrane was incubated with an HRP-conjugated secondary antibody (1:1,000, Sigma-Aldrich) for 1 h, followed by washes with TBS-T. Immunoreactivity was detected with an enhanced chemiluminescence detection kit (Pierce, Rockford, IL, USA). The exposure time was adjusted according to the fluorescence intensity, and the images were developed and fixed. Densitometric assessment of the bands on the autoradiogram was performed using with RS Image Pro v.4.5 (Roper Scientific, Trenton, NJ, USA).

### Inhibitory Avoidance (IA) Training

Inhibitory avoidance (IA) training is a hippocampus-dependent form of single-trial learning in which a rodent avoids entering a dark arena where it has previously received a foot shock. The IA apparatus (Institute of Biomedical Engineering, Tianjin, China) is a trough-shaped alley (15 cm deep, 20 cm wide at the top, and 6.4 cm wide at the floor) consisting of a 30-cm long safe compartment and a 38-cm long shock compartment separated by a retractable door. The safe compartment was white and illuminated and the shock compartment was black and dark. Foot shocks were delivered to the grid floor of the shock chamber *via* a constant current scrambler circuit. The apparatus was located in a sound-attenuated, nonilluminated room. During training sessions, rats in the second cohort were individually placed in the illuminated compartment of the IA apparatus with its head facing away from the door 1 h after the last injection. When the animal turned to face the door, the door was lifted to reveal the dark-shock compartment. Once the rat was fully inside the dark compartment, the door was closed and a single foot shock (0.4 mA for 2 s) was delivered. The rat was then returned to its home cage and tested for memory retention at 24 h after this training session. The rats were then placed in the illuminated compartment of the apparatus facing away from the door. After turning 180°, the door was lifted, the timer started, and the latency to enter the dark compartment with all four paws (maximum 600 s) was recorded and used as the index of retention.

### Quantitative Real-Time Reverse Transcription-PCR

After IA training, rats were immediately deeply anesthetized with sodium pentobarbital (100 mg/kg, i.p.) and transcardially perfused with 100 ml saline solution. Hippocampi were immediately dissected on ice. Samples of hippocampal tissue and primary hippocampal neurons were collected for quantitative real-time PCR. Real-time PCR was carried out according to our previous protocol (Yu W. et al., 2015). Briefly, total RNA from cultured neurons and hippocampi were extracted using TRIzol reagent (Invitrogen, Carlsbad, CA, USA) and reverse transcribed using the Perfect Real Time PrimeScript RT Reagent kit (Takara Bio, Japan). Expression levels of BDNF and GAPDH (control) mRNA were quantified on a Light Cycler system (Roche, Indianapolis, IN, USA) using the QuantiTect SYBR Green PCR kit (Qiagen, Valencia, CA, USA). The sequences of forward and reverse primers are listed in Table 1. The expression level of each gene was normalized to the mean Ct value of the housekeeping gene GAPDH in the PCR array. Differences in expression between treatment groups were determined with the 2^−ΔΔCt^ method. Each sample was analyzed in triplicate.

**Table 1:** The sequences of gene-specific primers used for quantitative Real-Time Reverse Transcription-PCR (qRT-PCR).

Gene name	Forward (5′–3′)	Reverse (5′–3′)
BDNF	GTCACAGCGGCAGATAAAAAG	ATGGGATTACACTTGGTCTCGT
GAPDH	AGGGTGGTGGACCTCATGG	AGCAACTGAGGGCCTCTCTCTT

### Statistical Analysis

The results are expressed as mean ± SEM. A two-tailed *t*-test for independent samples was used for two-group comparisons. One-way analysis of variance followed by the Newman-Keuls multiple comparison tests were used to compare the control and treatment groups. *P* < 0.05 was considered statistically significant.

## Results

### MK-801 Suppresses BDNF mRNA Expression and Protein Secretion in Primary Cultured Hippocampal Neurons

**Figure 1:**
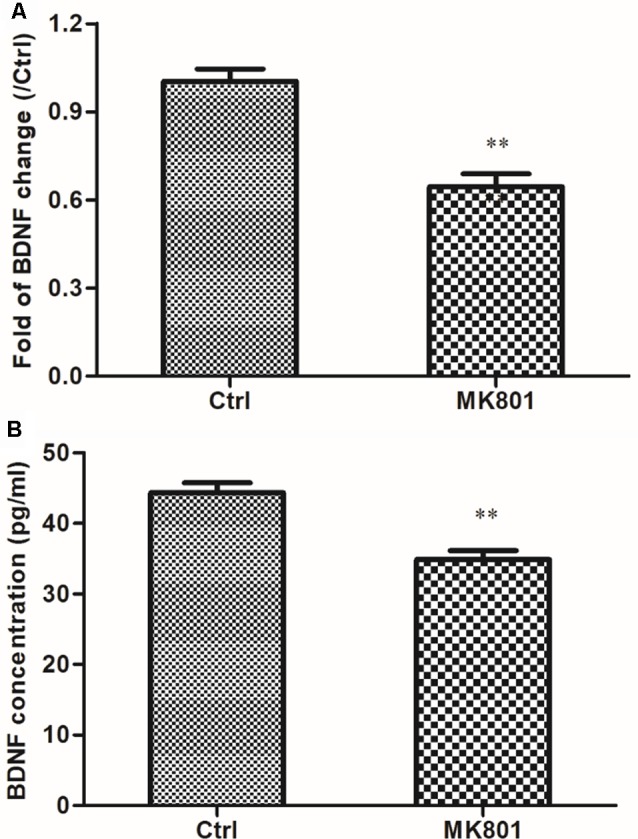
MK-801 suppresses brain-derived neurotrophic factor (BDNF) mRNA expression and protein secretion in primary cultured hippocampal neurons. **(A,B)** Hippocampal neurons were cultured in the presence or absence of 20 μM MK-801 for 24 h and BDNF mRNA and protein levels in cell lysates and culture supernatant were measured by quantitative real-time (qRT-PCR; **A**) and enzyme-linked immunosorbent assay (ELISA; **B**), respectively. Densitometric values are expressed as mean ± SEM of three independent experiments. **P < 0.01 vs. control group (Student’s *t*-test).

The effect of MK801 on BDNF mRNA expression in primary cultured hippocampal neurons following treatment with 20 μM MK-801 for 24 h was assessed by quantitative real-time PCR. BDNF mRNA level in the neurons was reduced to 65% of the control by the treatment (*t* = 5.964, *df* = 4, *P* = 0.004; Figure 1A; Supplementary Table S4). To determine whether MK801 treatment also blocked BDNF protein secretion, we measured the protein level in the supernatant of neuronal cultures by ELISA and found that it was lower in cultures treated with 20 μM MK-801 than in the control group (34.9 ± 1.2 vs. 44.3 ± 1.4 pg/ml; *t* = 5.041, *df* = 4, *P* = 0.007; Figure 1B; Supplementary Table S3).

### Risperidone Reverses the MK801-Induced Decrease in BDNF mRNA Expression and Protein Secretion in Hippocampal Neurons

**Figure 2:**
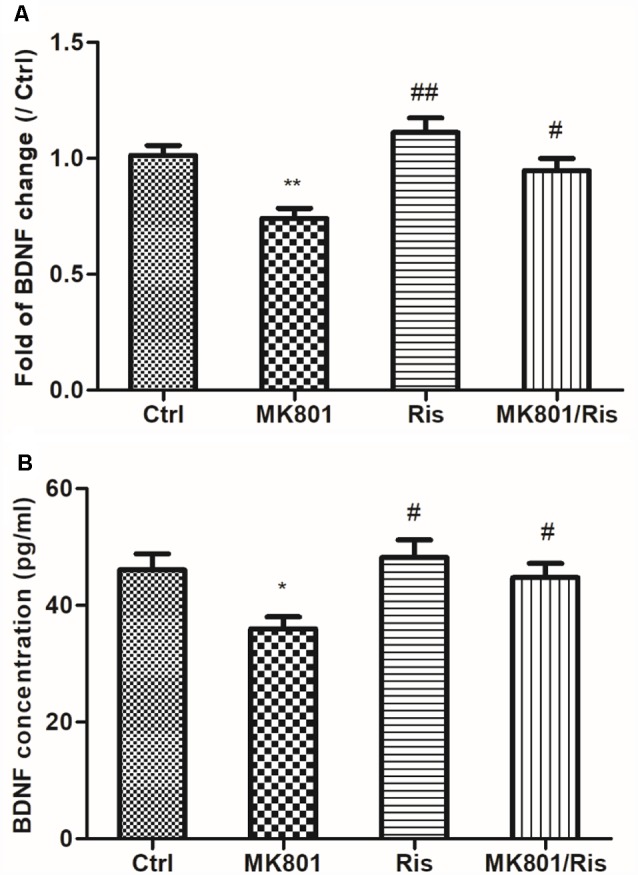
Risperidone reverses MK801-induced decreases in BDNF mRNA expression and protein secretion in hippocampal neurons. **(A,B)** Cultured neurons were treated with or without 20 μM MK801 for 2 h; the culture medium was then replaced by a fresh medium with or without 10 μM risperidone for 24 h. BDNF mRNA and protein levels in cell lysates and culture supernatant were evaluated by quantitative real-time PCR **(A)** and ELISA **(B)**, respectively. Densitometric values are expressed as the mean ± SEM of three independent experiments. *P < 0.05, **P < 0.01 vs. control group; ^#^*P* < 0.05, ^##^*P* < 0.01 vs. MK801 group.

BDNF mRNA expression in primary cultured hippocampal neurons incubated with or without 20 μM MK-801 for 2 h with or without 10 μM risperidone for 24 h was assessed by quantitative real-time PCR. BDNF transcript level was reduced to 74% of the control by 20 μM MK-801 treatment for 2 h (*F* = 9.560, *df1* = 3, *df2* = 8, *P* = 0.005), and was restored to about 95% of the control after 10 μM risperidone treatment for 24 h, which was higher than the level in the MK801 group (*P* = 0.021; Figure 2A; Supplementary Table S7). BDNF concentration in culture supernatant was reduced to 36.0 ± 2.0 (pg/ml) for neurons treated with 20 μM MK-801 for 2 h (*F* = 4.387, *df1* = 3, *df2* = 8, *P* = 0.042), as determined by ELISA. However, the concentration was 44.8 ± 2.4 (pg/ml) after 10 μM risperidone treatment for 24 h, which was higher than the level in the MK801 group (*P* = 0.041; Figure 2B; Supplementary Table S6).

### MK-801 Treatment Inhibits BDNF Protein Expression in Rat Hippocampus

**Figure 3:**
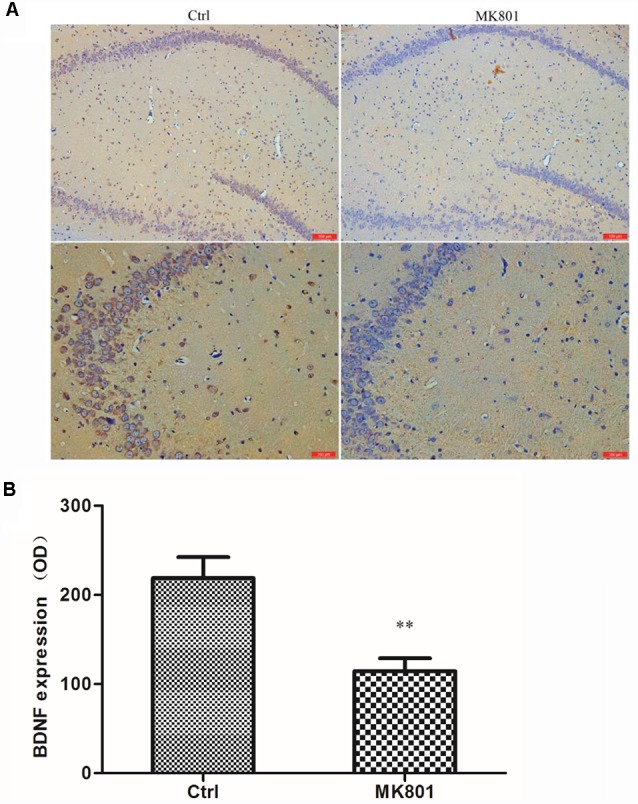
BDNF immunoreactivity in the hippocampus of MK-801-treated rats. MK-801 was administered by daily injection (0.5 mg/kg body weight, i.p.) for 6 days. Control (Ctrl) animals received an equal volume of normal saline. **(A)** Weak BDNF immunoreactivity was detected in the hippocampus of MK801-treated rats (*n* = 4 per group), while extensive positive staining was observed in the control group. Scale bar: 100 μm (upper) and 200 μm (under). **(B)** Computer-assisted image analysis revealed decreased BDNF-like immunoreactivity in MK-801-treated as compared to saline-treated rats. OD, optical density. Data represent mean ± SEM. Statistical differences between two groups were evaluated with the Student’s *t*-test. **P < 0.01 vs. control group.

**Figure 4:**
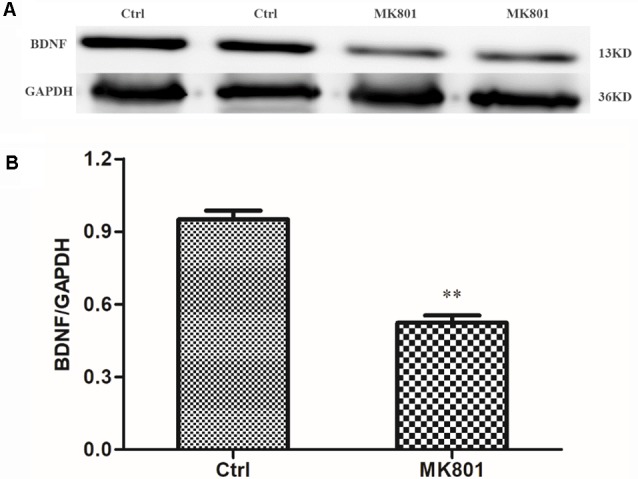
Western blot analysis of BDNF protein level in the hippocampus of MK-801-treated rats. MK-801 was administered daily by injection (0.5 mg/kg body weight, i.p.) for 6 days. Control animals received an equal volume of normal saline. **(A)** BDNF protein expression was detected by western blot analysis of hippocampal lysates from MK-801-treated (MK801) and control (Ctrl) rats (*n* = 4 per group). **(B)** Densitometric values are expressed as mean ± SEM relative to the loading control. Statistical differences between two groups were evaluated with the Student’s *t*-test. **P < 0.01 vs. control group.

BDNF levels in the hippocampus of rats with MK-801-induced schizophrenia (0.5 mg/kg daily for 6 days) were examined by immunohistochemistry and western blotting. BDNF-like immunoreactivity was lower in the MK-801-treated group than in saline-treated controls (115 ± 14 vs. 219 ± 24; *t* = 3.756, *df* = 6, *P* = 0.009), as determined by computer-assisted image analysis (Figure 3; Supplementary Table S1). This down-regulation was confirmed by western blot analysis of hippocampal tissue lysates; BDNF protein expression was reduced to 50% of control in MK-801-treated hippocampus (*t* = 8.894, *df* = 6, *P* < 0.001; Figure 4; Supplementary Figure S1, Table S2).

### Risperidone Reverses the Decrease in BDNF mRNA Expression in the Hippocampus of MK801-Treated Rats

**Figure 5:**
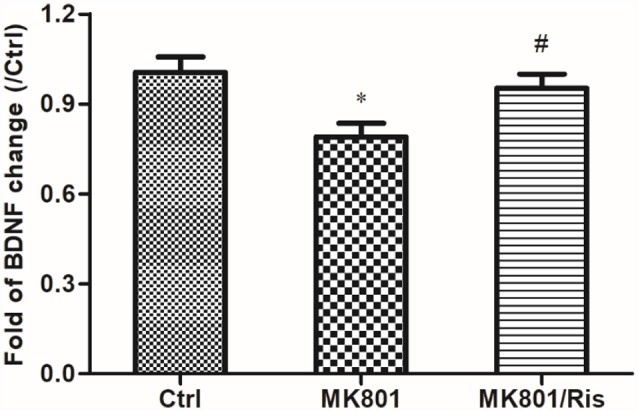
Risperidone reverses the downregulation of BDNF in the hippocampus of rats treated with MK801. Rats were administered MK-801 (0.5 mg/kg body weight, i.p.) once daily for 6 days. Subsequently, rats in the MK-801+ risperidone group were injected with risperidone (1 mg/kg body weight, i.p.) once daily for 7 days, whereas those in the MK-801 group received an equal volume of normal saline. Control group received an equal volume of normal saline once daily for 13 days. BDNF expression in hippocampal lysates was detected by real-time PCR (*n* = 4 per group). Densitometric values are expressed as mean ± SEM. *P < 0.05 vs. control group; ^#^*P* < 0.05 vs. MK801 group.

BDNF mRNA level in the hippocampus of rats was examined by quantitative real-time PCR. BDNF mRNA expression was decreased to 79% of the control after daily MK801 injection (0.5 mg/kg body weight, i.p.) for 6 days (*F* = 5.481, *df1* = 2, *df2* = 9, *P* = 0.028; Figure 4). However, the transcript level was restored to 95% of the control by risperidone injection (1 mg/kg body weight, i.p.) once daily for 7 days and was higher than that in MK-801-treated rats (*P* = 0.04; Figure 5; Supplementary Table S5), indicating that risperidone reversed the MK801-induced decrease in BDNF mRNA expression in the rat hippocampus.

### Risperidone Reverses Cognitive Impairment in MK801-Treated Rats

**Figure 6:**
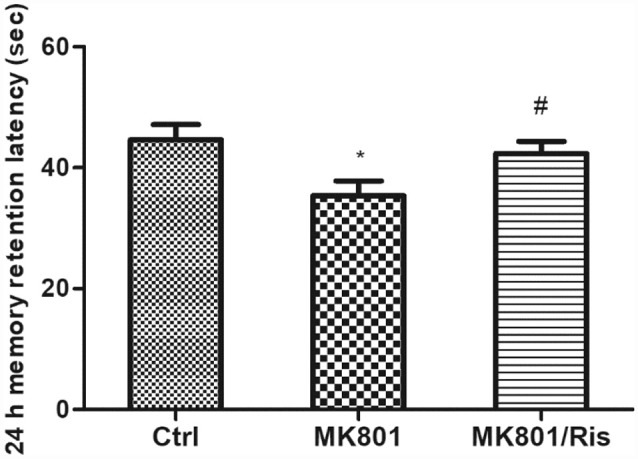
Risperidone reverses the MK801-induced decrease in retention latency in rats. MK-801 was administered by injection (0.5 mg/kg body weight, i.p.) once daily for 6 days. Subsequently, rats in the MK-801+ risperidone group were injected with risperidone (1 mg/kg body weight, i.p.) once daily for 7 days whereas the MK-801-only group received an equal volume of normal saline. Control group received an equal volume of normal saline once daily for 13 days. Retention latency was evaluated with the inhibitory avoidance (IA) test. Data are shown as mean ± SEM. *P < 0.05 vs. control group; ^#^*P* < 0.05 vs. MK801 group.

In the IA test, MK-801-treated rats showed a shortest latency to enter the darkened chamber 24 h after foot shock in three groups (Ctrl: 44.6 ± 2.5 s, MK-801: 35.4 ± 2.5 s, MK-801/Ris: 42.3 ± 2.0 s; *F* = 4.298, *df1* = 2, *df2* = 21, *P* = 0.027; Figure 6), indicating that repeated administration of MK-801 abolished hippocampus-dependent learning. However, risperidone prolonged the latency to 42.3 ± 2.0 s relative to MK-801-treated rats (*P* = 0.045; Figure 6; Supplementary Table S8). Thus, risperidone reverses MK801-induced cognitive impairment.

## Discussion

The results of the present study demonstrate that repeated high doses of MK-801 inhibited BDNF expression in the rat hippocampus, with a concomitant decline in cognitive function. MK-801 is a non-competitive NMDA-R antagonist that has potent psychomimetic effects such as hallucinations and psychomotor symptoms and has therefore been used extensively in schizophrenia research. MK-801 treatment increases NMDA-r expression at low doses but has the opposite effect at high concentrations (Xi et al., 2011). Repeated high doses of MK-801 (0.5 mg/kg) can mimic some of the behavioral characteristics (e.g., hyperlocomotion and disruption of prepulse inhibition) and neurochemical changes associated with schizophrenia in animal models (Eyjolfsson et al., 2006). Chronic rather than acute administration of NMDA-R antagonists either in the early period of central nervous system development or in adulthood is considered to produce a better model since it causes cellular and molecular alterations in the brain that are similar to those observed in schizophrenia patients (Blot et al., 2013). Our observations that MK-801 treatment reduced BDNF mRNA and protein levels in cognition-related brain areas such as the hippocampus in rats are consistent with the reduction in cerebrospinal fluid BDNF levels reported in patients (Pillai et al., 2010).

BDNF is the most abundant neurotrophin in the brain and affects developmental processes including neuro-, glio-, and synaptogenesis; provides neuroprotection; and controls transient and long-lasting synaptic interactions that influence memory and cognition (Kowiański et al., 2018). By binding to TrkB and p75NTR receptors, BDNF mediates LTP and LTD, respectively, which are two forms of synaptic plasticity representing the cellular correlates of learning and memory in the hippocampus (Lu and Martinowich, 2008). The binding of BDNF to the TrkB receptor triggers its homodimerization and autophosphorylation of intracellular tyrosine residues (Kaplan and Miller, 2000). The BDNF/TrkB receptor complex activates various signaling molecules including mitogen-activated protein kinase (MAPK), phosphatidylinositol 3-kinase, phospholipase C-γ, and guanosine triphosphate hydrolases (GTPases) of the Ras homolog (Rho) family (Kowiański et al., 2018) that modify the function of local synaptic targets and also have long-term effects on gene transcription. The MAPK pathway is critical for dendritic growth and branching in hippocampal neurons (Kwon et al., 2011). BDNF/TrkB complex-induced activation of GTPases stimulates actin and microtubule synthesis, which results in the growth of neuronal fibers (Gonzalez et al., 2016). Deficits in these synaptoplastic processes may underlie some of the cognitive deficits exhibited by patients with schizophrenia, which is closely related to imbalanced circuit-level expression of BDNF signaling molecules (Autry and Monteggia, 2012).

Our previous study showed that MK-801 increased BDNF expression levels in hippocampal astrocytes *in vitro*. Since BDNF is expressed in both neurons and astrocytes of the hippocampus, the contribution of BDNF signaling by hippocampal neurons is unclear. We used primary neuronal cultures to evaluate the direct effects of MK-801 on hippocampal neurons. Consistent with our *in vivo* results, MK-801 reduced BDNF levels in these neurons *in vitro*.

The subunit composition of NMDA-Rs may explain the differences in MK-801 induced BDNF expression between astrocytes and neurons. NMDA-Rs are a family of ionotropic glutamate receptors that comprise homologous subunits selected from three sub-families with multiple members: GluN1 (with eight alternatively spliced isoforms), GluN2 (four subtypes, A–D), and GluN3 (two subtypes, A and B; Fedele et al., 2018). The most common composition of functional NMDA-Rs includes two obligatory NR1 subunits and two regionally localized NR2 subunits, two GluN1, or one GluN2 and one GluN3 subunit. NMDA-Rs composed of GluN1 and GluN3 subunits are also functional but lack the glutamate/NMDA binding site (Smothers and Woodward, 2007). The different subunits define the pharmacological and kinetic properties of the receptor such as conductance, deactivation time, sensitivity to Mg^2+^, and antagonist specificity (Akazawa et al., 1994). Activated NMDA-Rs are strongly permeable to Ca^2+^, Na^+^, and K^+^; however, permeability to certain ions is highly dependent on subunit composition—e.g., the presence of the GluN3 subunit increases permeability to Mg^2+^ while reducing Ca^2+^ permeability and overall current flow (Cavara and Hollmann, 2008). NMDA-R subunits in glial cells have characteristics that are different from those in neurons (e.g., the absence of Mg^2+^ block and reduced Ca^2+^ permeability; Dzamba et al., 2013). Ca^2+^ influx through NMDA-Rs plays a key role in synaptic transmission, neuronal development, and plasticity (Salami and Fathollahi, 2000). Glutamate-evoked increases in Ca^2+^ influx are inhibited by MK-801 and the selective NR2B antagonists, ifenprodil and Ro25-6981, in neurons but not in astrocytes (Kato et al., 2006). In addition to the binding of glutamate to the GluN2 subunit and of either glycine or D-serine to the GluN1 subunit at the glycine modulatory site, the neuron must be simultaneously depolarized, which relieves the Mg^2+^ blockade of the channel (Balu and Coyle, 2015). Activated NMDA-R allows Ca^2+^ entry into neurons, which triggers a cascade of intracellular events that mediate local, acute functional synaptic plasticity and changes in gene expression that influence neural structure over the long term (Balu and Coyle, 2015). However, MK-801 non-competitively inhibits glutamate uptake and also induces dose-dependent and reversible depolarization in astrocytes (Longuemare et al., 1996), thereby activating voltage-sensitive L-channels for Ca^2+^, which leads to a rise in free cytosolic Ca^2+^ and alters BDNF expression (Zhu et al., 2012). In addition, activation of γ-aminobutyric acid (GABA) A receptor in neurons induces an influx of Cl^−^ through the receptor pore, leading to hyperpolarization; however, astrocytes become slightly depolarized following exposure to GABAA receptor agonists (Zhu et al., 2012). This suggests that MK-801 modulates BDNF expression in astrocytes and neurons *via* distinct mechanisms.

Our study also showed that risperidone reverses MK801-induced cognitive impairment in rats. The mechanism underlying the improvement in cognitive function in patients with schizophrenia by risperidone is not well understood. In our study, risperidone reversed the MK801-induced decrease in BDNF expression in the hippocampus of rats, which is consistent with a previous report (Chen et al., 2017). Our work lacked the risperidone alone group in the *in vivo* system, but there are some works that have found that risperidone can evoke a statistically significant increase in the level of BDNF in the hippocampus (Yu B. et al., 2015; Rogóż et al., 2017). The same was observed in cultured hippocampal neurons treated with MK801. Thus, risperidone may alleviate MK801-induced cognitive impairment in rats involving upregulation of BNDF signaling in the hippocampus.

Risperidone is a SGA with high affinity for both serotonin 2A and dopamine D2 receptors and a modest affinity for histamine and α2 adrenergic receptors (Kumari et al., 2015). Previous studies reported that cognitive impairment induced by repeated NMDA-R blockade can be reversed by SGAs but not by first-generation antipsychotic drugs (Song et al., 2016; Liu et al., 2018). However, risperidone did not reverse MK-801-induced cognitive deficits in the standard water maze and radial arm maze tasks (Enomoto et al., 2008), while MK-801-induced deficits in social recognition in rats were reversed by aripiprazole but not olanzapine, risperidone, or cannabidiol (Deiana et al., 2015). These contradictory findings may be explained by the different experimental designs (i.e., different drug doses and treatment durations) of those studies or by the distinct pharmacological profiles (i.e., D2 and 5-HT2 receptor occupancy and affinity) of antipsychotic drugs. For example, the binding affinities of haloperidol (Ki = 2.6) and risperidone (Ki = 3.77) to D2 receptors are much higher than that of clozapine (Ki = 210), while risperidone has a lower D2 receptor occupancy rate (Correll, 2010).

In conclusion, our findings indicate that risperidone may improve MK801-induced cognitive impairment in rats involving activation of BNDF signaling in hippocampal neurons. Additional studies are currently underway to clarify the differences in BDNF expression associated with NMDA-Rs between hippocampal neurons and astrocytes.

## Ethics Statement

This study was carried out in accordance with the recommendations of the guidelines of the Animal Care Committee of Laboratory Animals of Shanghai Jiao Tong University School of Medicine and guidelines of the US National Institutes of Health Guide for the Care and Use of Laboratory Animals (Publication no. 80-23, revised in 1996). The protocol was approved by the Animal Care Committee of Laboratory Animals of Shanghai Jiao Tong University School of Medicine.

## Author Contributions

WY, JZ, WB, YW, HuZ, JX, HaZ and HL were responsible for designing and organizing the project and prepared the manuscript. WY, MZ, HF, HaZ, and LY performed the experiments and analyzed the data. All of the authors discussed the results and provided commentary on the manuscript.

## Conflict of Interest Statement

The authors declare that the research was conducted in the absence of any commercial or financial relationships that could be construed as a potential conflict of interest.
