# Supplementary material for: Risperidone Reverses the Downregulation of BDNF in Hippocampal Neurons and MK801-Induced Cognitive Impairment in Rats
Source: Front Behav Neurosci. 2019 Jul 23;13:163. doi: 10.3389/fnbeh.2019.00163 (PMC6664152; doi:10.3389/fnbeh.2019.00163)
Supplement: Supplementary file 1 [file Table_1.DOCX]

**Supplementary information**

**S1 Table. The data of BDNF immunoreactivity in rat hippocampus**

| BDNF | OD | |
| --- | --- | --- |
|  | Ctrl | Mk801 |
|  | 278 | 105 |
|  | 232 | 82 |
|  | 168 | 151 |
|  | 197 | 120 |

MK-801 was administered by daily injection (0.5 mg/kg body weight, i.p.) for 6 days. Control (Ctrl) animals received an equal volume of normal saline. BDNF immunoreactivity was detected in the hippocampus. Computer-assisted image analysis revealed BDNF-like immunoreactivity. Images were converted to grayscale, and optical density (OD) was analyzed with RS Image Pro v.4.5

**S2 Figure. Western blot analysis of BDNF in rat hippocampus**


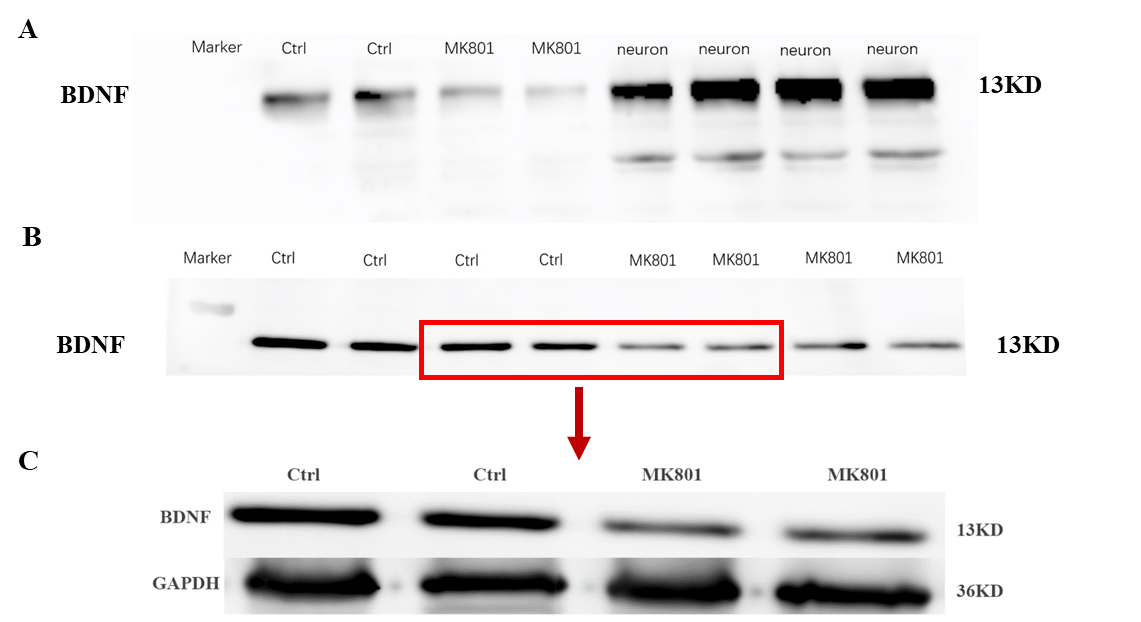


MK-801 was administered daily by injection (0.5 mg/kg body weight, i.p.) for 6 days. Control animals received an equal volume of normal saline. BDNF protein expression was detected by western blot analysis of hippocampal lysates. Images were developed and fixed. Densitometric assessment of the bands on the autoradiogram was performed using with RS Image Pro v.4.5. The neurons in A are primary hippocampal neurons, which are used as a reference for detecting BDNF. The bands in the red box in figure B are used in figure C.

**S3 Table. The data of BDNF protein in rat hippocampus by western blotting**

| BDNF | OD | |
| --- | --- | --- |
|  | Ctrl | Mk801 |
|  | 3784 | 1625 |
|  | 3193 | 1799 |
|  | 3265 | 2124 |
|  | 3502 | 2018 |

MK-801 was administered daily by injection (0.5 mg/kg body weight, i.p.) for 6 days. Control animals received an equal volume of normal saline. Images were developed and fixed. Densitometric assessment of the bands on the autoradiogram was performed using with RS Image Pro v.4.5

**S4 Table. The data of** **BDNF concentration in neurons induced by MK801**

| BDNF | pg/ml | |
| --- | --- | --- |
|  | Ctrl | Mk801 |
|  | 42.2 | 32.9 |
|  | 43.8 | 34.6 |
|  | 47.0 | 37.2 |

Hippocampal neurons were cultured in the presence or absence of 20 μM MK-801 for 24 h and protein levels culture supernatant were measured by ELISA.

**S5Table. The data of BDNF** **mRNA in neurons induced by MK801**

| BDNF Ctrl Mk801 | | |
| --- | --- | --- |
|  | 0.953265 | 0.656176 |
|  | 1.085847 | 0.713846 |
|  | 0.971572 | 0.564169 |

Hippocampal neurons were cultured in the presence or absence of 20 μM MK-801 for 24 h and BDNF mRNA in cells were measured by quantitative real-time PCR.

**S6 Table. The data of BDNF mRNA in rat hippocampus induced by MK801 or Ris**

| BDNF | Ctrl | Mk801 | MK801/Ris |
| --- | --- | --- | --- |
|  | 1.134054 | 0.8970652 | 1.084787 |
|  | 0.901647 | 0.6835092 | 0.950722 |
|  | 1.033143 | 0.7602526 | 0.863827 |
|  | 0.953143 | 0.8202526 | 0.910256 |

Rats were administered MK-801 (0.5 mg/kg body weight, i.p.) once daily for 6 days. Subsequently, rats in the MK-801+ risperidone group were injected with risperidone (1 mg/kg body weight, i.p.) once daily for 7 days, whereas those in the MK-801 group received an equal volume of normal saline. Control group received an equal volume of normal saline once daily for 13 days. BDNF mRNA expression in hippocampal lysates was detected by quantitative real-time PCR.

**S7 Table. The data of BDNF concentration in neurons induced by MK801 or Ris**

| BDNF | pg/ml | | | |
| --- | --- | --- | --- | --- |
|  | Ctrl | Mk801 | Ris | MK801/Ris |
|  | 44.3 | 36.6 | 43.2 | 41.1 |
|  | 51.5 | 39.1 | 47.8 | 49.3 |
|  | 42.4 | 32.2 | 53.5 | 43.9 |

Cultured neurons were treated with or without 20 μM MK801 for 2 h; the culture medium was then replaced by fresh medium with or without 10 μM risperidone for 24 h. BDNF protein levels in culture supernatant were evaluated by ELISA.

**S8 Table. The data of BDNF mRNA in neurons induced by MK801 or Ris**

| BDNF | Ctrl | Mk801 | Ris | MK801/Ris |
| --- | --- | --- | --- | --- |
|  | 1.074054 | 0.827065 | 0.992235 | 1.034787 |
|  | 0.931634 | 0.683509 | 1.204421 | 0.950722 |
|  | 1.033143 | 0.710252 | 1.139739 | 0.855827 |

Cultured neurons were treated with or without 20 μM MK801 for 2 h; the culture medium was then replaced by fresh medium with or without 10 μM risperidone for 24 h. BDNF mRNA levels in cells were evaluated by quantitative real-time PCR.

**S9 Table. The data of 24 h memory retention latency (sec)**

| Ctrl | MK801 | MK801/Ris |
| --- | --- | --- |
| 33 | 25 | 35 |
| 39 | 28 | 37 |
| 42 | 32 | 38 |
| 43 | 35 | 42 |
| 46 | 37 | 43 |
| 47 | 38 | 47 |
| 52 | 42 | 45 |
| 55 | 46 | 52 |

MK-801 was administered by injection (0.5 mg/kg body weight, i.p.) once daily for 6 days. Subsequently, rats in the MK-801+ risperidone group were injected with risperidone (1 mg/kg body weight, i.p.) once daily for 7 days whereas the MK-801-only group received an equal volume of normal saline. Control group received an equal volume of normal saline once daily for 13 days. Retention latency was evaluated with the IA test.
